# Tumor tissue-associated *Phascolarctobacterium* is associated with lymph node metastasis, prognosis, and immune-contexture features in colorectal cancer

**DOI:** 10.3389/fcimb.2026.1784151

**Published:** 2026-07-08

**Authors:** Jiale Li, Xiyue Hu, Yunxiao Liu, Jian Ma, Laifeng Ren, Jinyuan Guo, Xin Zhang, Yongsheng Meng, Jing Liu, Jiaqi Zhao, Likun Zan, Xu Guan, Wenqi Bai

**Affiliations:** 1 Department of Colorectal Surgery, Shanxi Province Cancer Hospital/Hospital Affiliated to Cancer Hospital, Chinese Academy of Medical Sciences/Cancer Hospital Affiliated to Shanxi Medical University, Taiyuan, China; 2 Department of Colorectal Surgery, National Cancer Center/National Clinical Research Center for Cancer/Cancer Hospital, Chinese Academy of Medical Sciences and Peking Union Medical College, Beijing, China; 3 Department of Colorectal Cancer Surgery, The Second Affiliated Hospital of Harbin Medical University, Harbin, Heilongjiang, China; 4 Central Laboratory, Shanxi Province Cancer Hospital/Shanxi Hospital Affiliated to Cancer Hospital, Chinese Academy of Medical Sciences/Cancer Hospital Affiliated to Shanxi Medical University, Taiyuan, Shanxi, China; 5 The Second Clinical Medical School, Shanxi Medical University, Taiyuan, Shanxi, China; 6 Department of Tumor Biobank, Shanxi Cancer Hospital/Shanxi Hospital Affiliated to Cancer Hospital, Chinese Academy of Medical Sciences/Cancer Hospital Affiliated to Shanxi Medical University, Taiyuan, Shanxi, China; 7 School of Basic Medicine, Shanxi Medical University, Taiyuan, Shanxi, China; 8 Department of Pathology, Shanxi Cancer Hospital/Shanxi Hospital Affiliated to Cancer Hospital, Chinese Academy of Medical Sciences/Cancer Hospital Affiliated to Shanxi Medical University, Taiyuan, Shanxi, China

**Keywords:** colorectal cancer, immune-contexture features, lymph node metastasis, *Phascolarctobacterium*, tumor tissue-associated microbiome

## Abstract

**Background:**

Lymph node metastasis (LNM) critically influences prognosis in colorectal cancer (CRC), yet the mechanisms driving this process, particularly the contribution of the intratumoral microbiota, remain insufficiently defined.

**Methods:**

We performed 16S rRNA sequencing on tumor tissue from a discovery cohort of 122 CRC patients, followed by validation in one internal and one external validation cohort. Immunohistochemistry (IHC), fluorescence *in situ* hybridization (FISH), and transcriptomic deconvolution were used to explore tumor immune-contexture features and tissue-associated *Phascolarctobacterium*-like signals. Bulk RNA-seq data were analyzed using Weighted Gene Co-expression Network Analysis (WGCNA) and pathway enrichment to explore host transcriptomic modules and molecular pathways associated with microbial abundance.

**Results:**

Higher tumor tissue-associated Phascolarctobacterium abundance showed a modest positive association with lymph node metastasis and was associated with worse overall survival in the discovery cohort (HR = 3.892, 95% CI = 1.441–10.513, P = 0.007). These tumors showed exploratory immune-contexture differences, including lower CD8+ T-cell-related signals and higher macrophage/M2 macrophage-related signals. WGCNA identified exploratory abundance-associated modules enriched in keratinocyte differentiation, epithelial development, and MAPK signaling, whereas low-abundance-associated modules were linked to lipid metabolism and redox regulation.

**Conclusions:**

Tumor tissue-associated *Phascolarctobacterium* abundance showed exploratory associations with lymph node metastasis, poorer overall survival in the discovery cohort, and immune-contexture features in CRC. These findings are exploratory and require validation in larger independent cohorts and functional studies.

## Introduction

Colorectal cancer (CRC) is among the three most prevalent malignancies worldwide, accounting for more than 900,000 deaths annually according to GLOBOCAN 2020 data (Sung et al., 2021). Recent analyses of the global burden ofCRC from 1990 to 2021 revealed heterogeneous trajectories across regions and predicted persistent disparities through 2040 (Chen et al., 2025). As with many malignancies, global cancer trends are closely influenced by diet, lifestyle, and environmental exposures (Bahrami and Tafrihi, 2023). Lymph node metastasis constitutes a key pathway ofCRC spread and portends unfavorable outcomes (Argilés et al., 2020); nevertheless, the mechanisms driving nodal involvement are not yet fully elucidated. Against this backdrop, clarifying the biological drivers of tumor progression and metastasis is of particular importance, with the gut microbiota attracting increasing attention for its potential role in modulating the tumor microenvironment.

The gut microbiota is involved in the pathogenesis ofCRC and has drawn increasing attention in recent years; given its reported associations with the tumor microenvironment and disease progression, it is necessary to further elucidate its specific mechanisms related to metastasis (Takiishi et al., 2017; Ghosh et al., 2021; Wong and Yu, 2023). Under physiological conditions, commensal microbes contribute critically to the maintenance of intestinal barrier integrity and mucosal homeostasis, thereby supporting normal host physiology and immune regulation (Natividad and Verdu, 2013). Dysbiosis of the gut microbiota has emerged as a critical environmental factor in CRC development, promoting tumor initiation and progression via multiple mechanisms (Helmink et al., 2019). Specific bacterial species can release antigens or metabolites that induce mitochondrial reactive oxygen species (ROS), leading to DNA damage and activation of oncogenic pathways (Hosainzadegan et al., 2020). Enterotoxigenic Bacteroidesfragilis (ETBF) induces Th17-mediated inflammation and genotoxic stress (Dickson, 2018). When the gut microbial ecosystem is disrupted, pathogenic species and harmful metabolites can interfere with host physiology and facilitate CRC development (Li et al., 2022). In summary, microbe–host interactions are closely associated with CRC; however, their associations with the tumor microenvironment and lymph-node metastasis remain insufficiently characterized.

Prior studies have shown that *Fusobacterium nucleatum* is closely associated with the initiation, progression, and metastasis of CRC (Chen et al., 2020; Ou et al., 2022). Beyond these well-characterized CRC-associated bacteria, emerging evidence suggests that *Phascolarctobacterium* may also be linked to CRC. For example, long-term follow-up analyses of colorectal cancer screening attendees reported increased abundance of *Phascolarctobacterium succinatutens* in CRC and suggested an association with the timing of CRC diagnosis (Bucher-Johannessen et al., 2023). In addition to fecal screening cohorts, mucosal/tissue-associated microbiome studies have also reported increased ASVs classified as *Phascolarctobacterium* in CRC or adenoma-associated samples compared with healthy controls, suggesting that this genus may not be restricted to stool-based CRC signals (Senthakumaran et al., 2023). Recent species-resolved analyses further supported an association between *Phascolarctobacterium* and CRC and revealed distinctive community and functional features (Bucher-Johannessen et al., 2024). However, unlike *Fusobacterium nucleatum* or enterotoxigenic *Bacteroides fragilis*, mechanistic evidence for *Phascolarctobacterium* remains limited, and our study should therefore be viewed as an association-driven investigation focused on LNM and immune contexture.

Based on these insights, we conducted a multi-omics analysis integrating 16S rRNA sequencing, immunohistochemistry (IHC), and transcriptomic profiling in a cohort of 122 colorectal cancer surgical specimens, with further validation in one internal and one external validation cohort. This integrative approach was designed to systematically characterize tumor tissue-associated microbial features and their associations with lymph node metastasis and clinical outcomes.

Building on this rationale, we investigated whether tumor tissue-associated *Phascolarctobacterium* abundance was associated with lymph node metastasis, clinical outcomes, and local immune-contexture features. Specifically, we assessed whether tumors with higher *Phascolarctobacterium* abundance showed immune- contexture differences, including macrophage-related and T-cell-related features, characterized by increased macrophage infiltration — particularly M2-like macrophages — and reduced T-cell infiltration, including CD3+ and CD8+ T cells, whereas tumors with lower abundance showed higher T-cell- and B-cell-related signals, including CD8+ T-cell and memory B-cell signals. In parallel, we integrated bulk transcriptomic profiling with weighted gene co-expression network analysis (WGCNA) to identify host gene modules and pathways associated with Phascolarctobacterium abundance, with particular attention to immune-contexture association, epithelial programs, and metabolic and oxidative stress –related signatures.

## Methods

### Patient cohorts

This study included one discovery cohort, one internal validation cohort, and one external validation cohort of patients with histologically confirmed CRC. The discovery cohort comprised l22 patients who underwent curative surgical resection at Shanxi Province Cancer Hospital between 20l6 and 20l9. Clinicopathological data, including age of diagnosis, sex, tumor location, CEA, CAl9-9, peripheral blood immune cell composition (B cells, NK cells, T cells, and regulatory T cells), LNM status, and overall survival, were collected from medical records.

Validation Cohort l was derived from Shanxi Province Cancer Hospital, whereas Validation Cohort 2 was obtained from the Second Affiliated Hospital of Harbin Medical University. Survival analyses were performed exclusively in the discovery cohort. Survival status was defined as vital status at the last follow-up (0 = alive, 1 = deceased).

The discovery cohort and validation cohort 1 (both from Shanxi Province Cancer Hospital) were approved by the Biomedical Ethics Committee of Shanxi Province Cancer Hospital (Approval No. KY2023005). Validation cohort 2 was approved by the Institutional Review Board of the Second Affiliated Hospital of Harbin Medical University (Approval No. YJSKY2024-269). Written informed consent was obtained from all participants prior to inclusion in the study.

### Definition of tumor tissue samples

Intratumoral samples were defined as tissue specimens obtained from the tumor mass under direct visualization by an experienced pathologist. To minimize contamination from the intestinal lumen, samples were collected from the internal tumor tissue rather than the mucosal surface, avoiding visible luminal contents and mucus. Tumor tissues were sampled from regions enriched for malignant epithelial cells, as confirmed by routine histopathological evaluation.

### 16S rRNA sequencing

Microbial DNA was extracted from CRC tumor tissues using mechanical and enzymatic lysis, followed by purification with the QIAamp DNA Mini Kit (Qiagen). The V3–V4 hypervariable region of the 16S rRNA gene was amplified using primers 341F (5’-CCTACGGGNGGCWGCAG-3’) and 805R (5’-GACTACHVGGGTATCTAATCC-3’) with Phusion High-Fidelity PCR Master Mix (New England Biolabs). PCR conditions were as follows: 98 °C for 1 min; 30 cycles of 98 °C for 10 s, 50 °C for 30 s, and 72 °C for 30 s; followed by a final extension at 72 °C for 5 min.

PCR products were confirmed by 2% agarose gel electrophoresis, purified using AMPure XP beads (Beckman Coulter), quantified using a Qubit 3.0 fluorometer, and fragment integrity was assessed with an Agilent 2100 Bioanalyzer. Libraries were pooled at equimolar concentrations and sequenced on an Illumina NovaSeq 6000 platform (paired-end 250 bp).

Given that tumor tissues represent low-biomass samples, particular attention was paid to potential reagent- and environment-derived contamination during 16S rRNA sequencing. During DNA extraction, extraction blank controls were included by replacing tissue samples with sterile water and processing them using the same extraction reagents and protocols as biological samples. In addition, no-template controls (NTCs), in which sterile water was used as a template substitute, were included in each PCR amplification batch. In total, nine sterile water negative controls were included. All negative controls were subjected to the same library preparation and sequencing procedures as tissue samples.

In downstream analyses, potential contaminant ASVs were identified using the decontam R package with a prevalence-based approach, in which ASVs showing higher prevalence in negative controls than in biological samples were flagged as contaminants. ASVs classified as contaminants were removed prior to downstream analyses to minimize the impact of background contamination on microbiome profiling of low-biomass tumor tissues.

Raw sequencing reads were processed with QIIME2 (v2022.2). After removal of barcodes and primers, paired-end reads were quality filtered, denoised, merged, and screened for chimeras using the DADA2 plugin. Amplicon sequence variants (ASVs) were generated and taxonomically assigned within QIIME2 using the SILVA database (v138. 1). Per-sample sequencing quality metrics, including raw paired-end reads, merged reads, qualified reads, non-chimeric reads, Q20, Q30, GC content, read-retention rate, and final feature counts, are provided in Online Resource 3.

Representative ASVs were aligned to construct a phylogenetic tree. Alpha diversity (Shannon and Simpson indices) and beta diversity (non-metric multidimensional scaling [NMDS]) metrics were calculated to evaluate microbial richness and community structure using the vegan R package (v2.5.6). Group differences were assessed using permutational multivariate analysis of variance (PERMANOVA).

LEfSe analysis was performed at the genus level using the microbiomeMarker R package (v1.0.0) as an exploratory screening approach to identify candidate differentially enriched taxa. Statistical significance thresholds were set at P < 0.05 for both the Kruskal–Wallis and Wilcoxon tests, with a linear discriminant analysis (LDA) score > 2.0. Differentially abundant taxa were visualized using bar plots generated with ggplot2 (v3.4.0).

To account for the compositional nature of microbiome data, ANCOM-BC2 was additionally performed at the genus level as a sensitivity analysis. The ANCOM-BC2 results were used to assess whether candidate taxa identified by LEfSe showed directionally consistent abundance changes after compositionality-aware testing.

Because 16S rRNA analysis was limited to the genus level, we further refined taxonomic assignments by performing Basic Local Alignment Search Tool (BLAST) analysis on all ASVs annotated to Phascolarctobacterium. Most sequences matched Phascolarctobacterium succinatutens with high similarity. Based on this finding, we designed a species-specific fluorescence *in situ* hybridization (FISH) probe targeting Phascolarctobacterium succinatutens (5’-CAGCATCCTCATGCGAGATTGCT-3’, FITC-labeled; Exonbio, Guangzhou, China). The probe sequence was derived from the 16S rRNA gene of the dominant Phascolarctobacterium succinatutens strain in our dataset, and its specificity was confirmed via BLAST queries against reference sequences in the NCBI database.

### Association between microbial abundance and tumor immune infiltration

#### Prognostic stratification

In the discovery cohort, Phascolarctobacterium abundance was first analyzed as a log- transformed continuous variable in Cox proportional hazards regression. For exploratory cutoff-based visualization and sensitivity analyses, patients were dichotomized into high- and low-abundance groups using a ROC/Youden-derived cutoff based on survival status within the discovery cohort. This cutoff was used for exploratory Kaplan–Meier visualization and cutoff-based Cox analyses. To assess the robustness of the prognostic association, sensitivity analyses were performed. First, intratumoral Phascolarctobacterium abundance was modeled as a log-transformed continuous variable in a Cox proportional hazards model. Second, bootstrap resampling (1,000 iterations) was used to evaluate the stability of the ROC signal and the Youden-derived cutoff.

#### Immune infiltration

CIBERSORT (R package v1.03) was used to deconvolute bulk RNA-seq TPM matrices and estimate the relative proportions of22 immune cell types. CIBERSORT was run with the LM22 signature matrix and 1,000 permutations.Only samples with CIBERSORT deconvolution P < 0.05 were retained for downstream comparisons. Differences in immune cell infiltration between groups were assessed using the Wilcoxon rank-sum test or Student’s t-test, as appropriate (two-sided P < 0.05).

### Transcriptome sequencing

Total RNA was extracted from CRC tumor samples using TRIzol reagent (Invitrogen, USA). RNA integrity was assessed by agarose gel electrophoresis, and purity was measured using a NanoPhotometer (IMPLEN, USA). RNA concentration was quantified using a Qubit 2.0 fluorometer (Thermo Fisher Scientific, USA), and integrity was further confirmed with an Agilent 2100 Bioanalyzer (Agilent Technologies, USA). Polyadenylated mRNA was enriched using oligo(dT) beads, fragmented randomly, and reverse-transcribed into first- and second-strand cDNA.

Sequencing libraries were prepared through end repair, A-tailing, adapter ligation (including UMI tags and sample indices), and PCR amplification. Library quality was evaluated using Qubit 2.0, Agilent 2100, and quantitative PCR (qPCR), and pooled according to the target sequencing depth for sequencing on the Illumina NovaSeq 6000 platform (paired-end 150 bp).

Raw reads were processed using CASAVA and quality-filtered to remove adapter sequences, poly-N regions, and low-quality reads, resulting in clean reads. Clean reads were aligned to the human reference genome GRCh38/hg38 using HISAT2 (v2.0.5). Gene expression levels were quantified using StringTie (v1.3.3b) and reported as fragments per kilobase million (FPKM), and raw read counts were generated using featureCounts for downstream differential expression analysis.

### FISH

Formalin-fixed, paraffin-embedded CRC tumor tissues were sectioned at 4 µm, deparaffinized, and subjected to proteinase K digestion at 37 °C for 20–30 min. After pre-hybridization, sections were incubated overnight at 42 °C with a Phascolarctobacterium succinatutens-specific probe (5′-CAGCATCCTCATGCGAGATTGCT-3 ′, FITC-labeled; Exonbio, Guangzhou, China). Post-hybridization washes were performed using SSC buffer under stringent conditions. Nuclei were counterstained with DAPI, and slides were mounted with anti-fade reagent. Fluorescence signals were visualized using an Olympus BX53 fluorescence microscope. The FISH probe targeting Phascolarctobacterium succinatutens was designed based on the 16S rRNA sequence of the predominant species within the Phascolarctobacterium genus identified in this study.

Species-specific FISH was performed to provide supportive spatial evidence for Phascolarctobacterium succinatutens-like signals in tumor tissue sections.

Representative high-magnification FISH images were quantified using QuPath 0.5.1. For each group, three individual tumor samples were included, and three representative tumor fields were selected from each sample within pathologist-reviewed tumor regions. Green-channel FISH-positive areas were measured within annotated tumor ROIs using a uniform threshold across all images. The FISH-positive area fraction was calculated as the green-channel FISH-positive area divided by the annotated tumor ROI area. The same thresholding parameters were applied to all images.

### IHC

Tissue microarrays (TMAs) comprising 30 CRC tumor samples, including 15 Phascolarctobacterium-high and 15 Phascolarctobacterium-low tumors, were stained following the Dako Cytomation Immunostain SP Kit protocol. Tissue sections were fixed in 4% neutral-buffered formalin, embedded in paraffin, and cut to 4 µm. After deparaffinization, rehydration, antigen retrieval, and blocking of endogenous peroxidase activity, sections were incubated with primary antibodies against CD3, CD8, and CD68. The antibody information was as follows: anti-CD3 antibody, mouse monoclonal antibody, clone LN10, Gene Tech, catalog no. GT2002, dilution 1:200; anti-CD8 antibody, rabbit monoclonal antibody, clone SP16, Gene Tech, catalog no. GT2112, ready-to-use; anti-CD68 antibody, mouse monoclonal antibody, clone KP1, Maixin Biotech, catalog no. KIT-0026, ready-to-use. Species-matched IgG controls were included as negative controls and are shown in Online Resource 16. Sections were subsequently incubated with secondary antibodies and developed using chromogenic detection. Slides were scanned at 20× using an Aperio AT2 scanner and analyzed in QuPath 0.5.1.

Immunohistochemical evaluation was restricted to pathologist-reviewed tumor regions identified by histomorphological features. Areas of necrosis, non-tumor tissue, mucosal tissue, and lamina propria were excluded from quantitative analysis. Tumor regions were manually annotated before image analysis. Tissue regions were detected using the “Simple Tissue Detection” module, and positive cells were counted using the “Positive Cell Detection” module with nuclear thresholds and staining intensity grading. For each marker, three representative high-power fields (40×) per sample were analyzed to obtain positive cell percentages within annotated tumor ROIs, calculated as the number of positive cells divided by the total number of detected cells. A representative workflow illustrating the exclusion of necrotic areas, non-tumor tissue, mucosal tissue/lamina propria, and the annotation of tumor regions for QuPath analysis is provided in Online Resource 17.

### WGCNA and functional enrichment

WGCNA was performed using the WGCNA R package (v1.72- 1) to identify co-expression modules associated with microbial abundance. Genes with low expression variance and outlier samples were removed. A soft-thresholding power (β = 5) was selected to construct a scale-free network. The adjacency matrix was transformed into a topological overlap matrix (TOM), followed by hierarchical clustering to detect gene modules (minimum module size = 100, merge cut height = 0. 1). Module eigengenes were correlated with *Phascolarctobacterium* abundance using Spearman’s correlation. Functional enrichment analysis of significant modules was performed using GO and KEGG pathways with the clusterProfiler R package (v4.4.4), applying thresholds of P < 0.05 and false discovery rate (FDR) < 0.05.

### Statistical analysis

Categorical variables were presented as n (%) and compared using Pearson’s χ² test or Fisher’s exact test. Continuous variables were expressed as mean ± standard deviation (SD) or median (interquartile range, IQR) and compared using Student’s t-test or the Wilcoxon rank-sum test, as appropriate. Survival analyses were performed using the Kaplan–Meier method and compared with the log-rank test. Statistical analyses were conducted using GraphPad Prism 10, R version 4.4.2, and QuPath 0.5.1. Two-sided P < 0.05 was considered statistically significant. Multivariate Cox proportional hazards regression was used to evaluate the association between Phascolarctobacterium abundance and overall survival after adjustment for selected clinical variables, including N stage and peripheral B-cell levels.

## Results

### Basic information and clinical characteristics of CRC patients

A total of 122 patients who underwent surgical resection for CRC were included in this study, comprising 77 patients with lymph node metastasis–negative (N0) disease and 45 patients with lymph node metastasis–positive (N+) disease. Baseline clinical characteristics were systematically collected for all patients, including sex, age, maximum tumor diameter (tumor size), serum carcinoembryonic antigen (CEA) levels, serum carbohydrate antigen 19-9 (CA19-9) levels, and peripheral blood immune cell profiles (T cells, B cells, regulatory T cells, and natural killer cells (NK cells).

As summarized in **Table 1**, there were no significant differences between the N0 and N+ groups in sex distribution (*P* = 0.218), age (*P* = 1.000), tumor size (*P* = 0.967), CEA (*P* = 0.798), CA19-9 (*P* = 0.982), or immune cell proportions (all *P* > 0.05), indicating that the two groups were generally balanced and comparable.

**Table 1:** Baseline clinical characteristics of CRC patients.

Characteristic	Overall (n=122)	N0 (n=77)	N+ (n=45)	P value
Sex, n (%)				0.218
Female	48 (39.3)	34 (44.2)	14 (31.1)	
Male	74 (60.7)	43 (55.8)	31 (68.9)	
T stage, n (%)				0.005
T1–T2	26 (21.3)	23 (29.9)	3 (6.7)	
T3–T4	96 (78.7)	54 (70.1)	42 (93.3)	
Status, n (%)				<0.001
0	105 (86.1)	74 (96.1)	31 (68.9)	
1	17 (13.9)	3 (3.9)	14 (31.1)	
Age (years), n (%)				1.000
≤65	75 (61.5)	47 (61.0)	28 (62.2)	
>65	47 (38.5)	30 (39.0)	17 (37.8)	
Tumor size (cm), n (%)				0.967
≤5	96 (78.7)	60 (77.9)	36 (80.0)	
>5	26 (21.3)	17 (22.1)	9 (20.0)	
B cell (%), median [IQR]	8.70 [6.20, 12.20]	9.60 [6.80, 12.40]	8.05 [6.00, 11.35]	
CEA (ng/mL), median [IQR]	2.08 [1.13, 4.53]	2.19 [1.08, 4.42]	1.86 [1.15, 4.70]	0.798
CA19-9 (U/mL), median [IQR]	11.55 [7.15, 19.74]	11.55 [7.98, 19.88]	11.32 [6.81, 17.94]	0.982
T cell (%), median [IQR]	68.40 [60.00, 74.40]	68.00 [60.00, 73.60]	70.65 [61.35, 75.50]	0.228
Treg (%), median [IQR]	5.40 [3.60, 6.90]	5.40 [4.00, 7.00]	5.40 [3.60, 6.90]	0.702
NK (%), median [IQR]	19.70 [14.70, 29.00]	22.10 [14.90, 29.50]	18.60 [14.10, 24.18]	0.153

CRC, colorectal cancer; N0, lymph node metastasis-negative; N+, lymph node metastasis-positive; tumor size: maximum tumor diameter; IQR, interquartile range; Status: 0 = alive, 1 = deceased.

In contrast, the proportion of patients with T3–T4 tumors was significantly higher in the N+ group than in the N0 group (93.3% vs. 70. 1%, *P* = 0.005), confirming the close association between advanced local invasion, lymph node metastasis, and adverse prognosis. The baseline characteristics of the internal validation cohort (n = 30) and external validation cohort (n = 36) were likewise comparable between N0 and N+ patients (all P > 0.05; Online Resources 1–2).

### Comparison of microbial composition between N0 and N+ CRC patients

**Figure 1:**
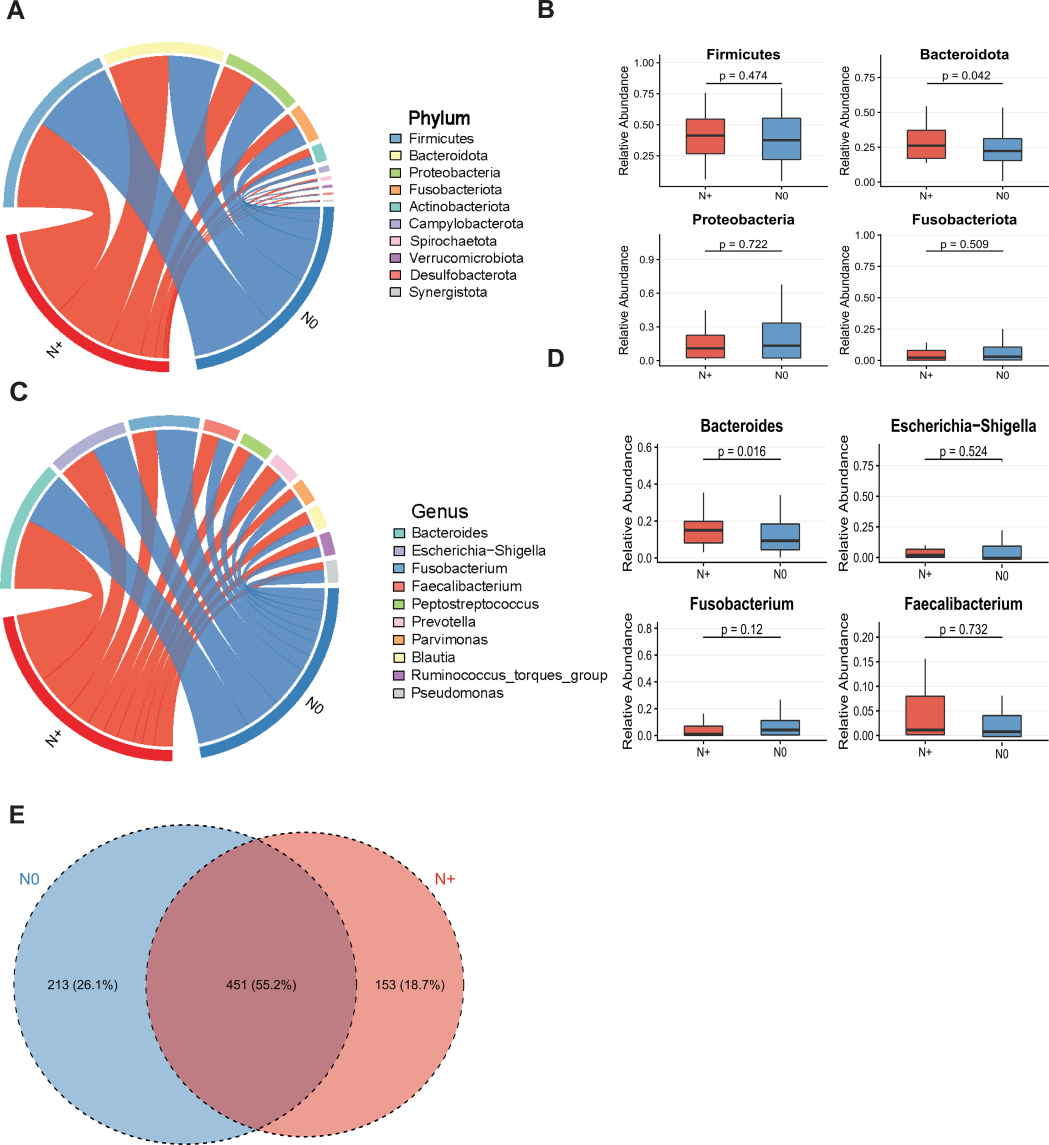
Microbial composition in CRC tumors. **(A)** Phylum-level chord diagram. **(B)** Boxplots of major phyla; Bacteroidota enriched in N +. **(C)** Genus-level chord diagram. **(D)** Boxplots of major genera; Bacteroides increased in N +. **(E)** Venn diagram showing shared and unique genera.

Sequencing quality was summarized for all tumor tissue samples included in the 16S rRNA sequencing analysis. Per-sample sequencing quality metrics, including raw paired-end reads, merged reads, quality-filtered reads, non-chimeric reads, Q20, Q30, GC content, read-retention rates, and final feature counts, are provided in Online Resource 3. We first characterized the intratumoral microbiome composition profiles in N0 and N+ patients. At the phylum level, *Firmicutes, Bacteroidota,Proteobacteria*, and *Fusobacteriota* were the dominant taxa in both groups, with Bacteroidota showing significant enrichment in the N+ group (*P* = 0.042) (**Figures 1A, B**). At the genus level, *Bacteroides*, *Fusobacterium*, *Escherichia–Shigella*, and *Faecalibacterium* were among the most abundant genera, of which *Bacteroides* was significantly increased in the N+ group (*P* < 0.05) (**Figures 1C, D**). Venn diagram analysis further demonstrated that most genera were shared between the N0 and N+ groups, whereas only a small subset of genera was detected exclusively in one group (**Figure 1E**). Although notable shifts in the abundance of *Bacteroidota* and *Bacteroides* were observed in this cohort, these patterns were not replicated in the internal and external validation cohorts, suggesting that tumor microbial taxonomic signals may be influenced by cohort-specific factors or limited sample size (Online Resources 4–5).

### Comparison of microbial diversity and differential abundance between N0 and N+ CRC patients

**Figure 2:**
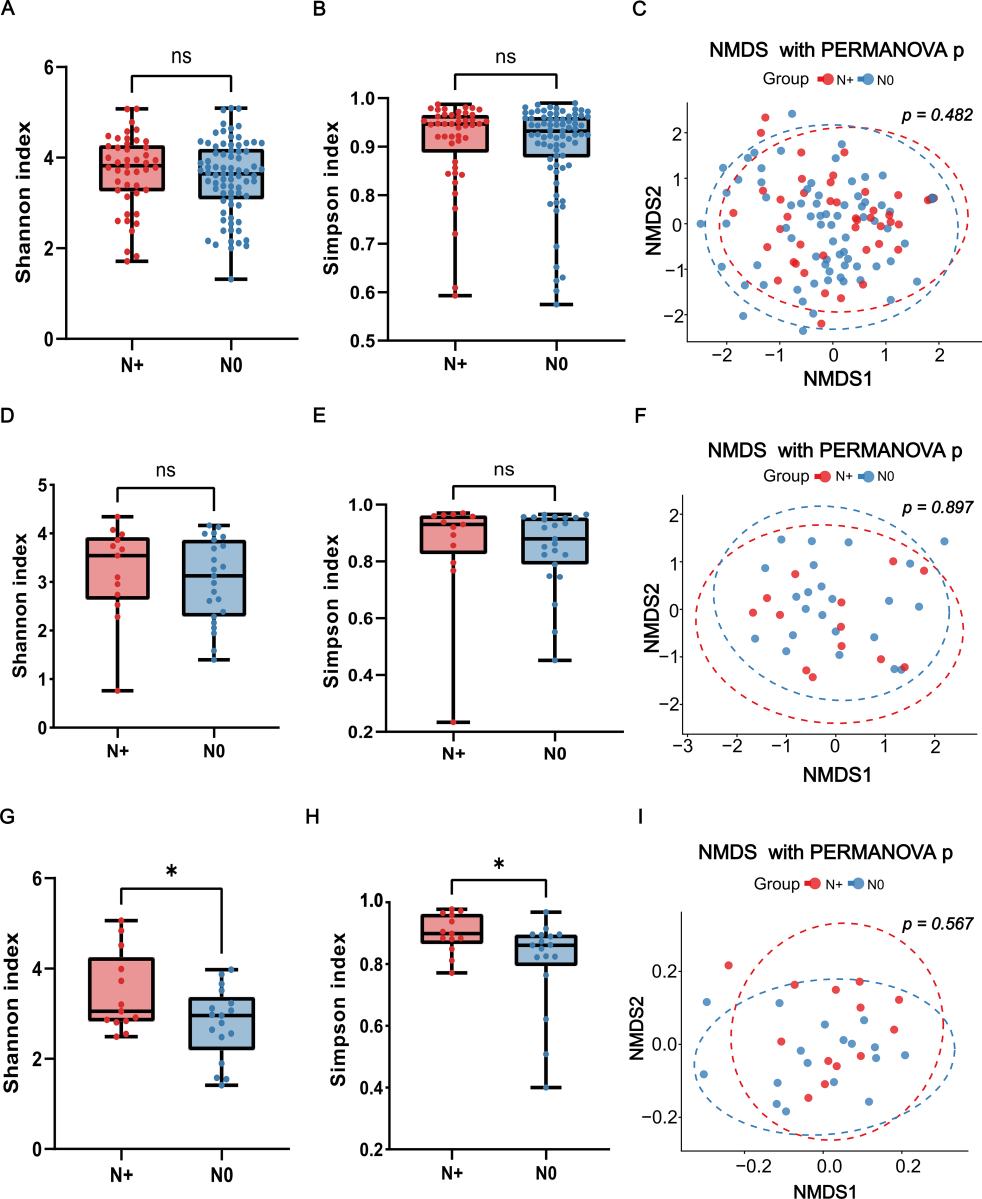
Diversity analysis and differential taxa in CRC tumors. **(A, B, D, E, G, H)** Alpha diversity (Shannon and Simpson indices); significant differences were observed in panels G–H (*P < 0.05*), while other panels showed no significant differences (*P > 0.05*). **(C, F, I)** Beta diversity evaluated by NMDS with PERMANOVA; no significant separation between N+ and N0 groups (all *P > 0.05*). Significance: *P < 0.05.

To evaluate microbial diversity in tumor tissues, α-diversity and β-diversity analyses were performed between the N0 and N+ groups. As shown in **Figures 2A, B**, two α-diversity indices, including the Shannon index (reflecting species richness and evenness) and the Simpson index (indicating community dominance and evenness), did not differ significantly between the groups (*P* > 0.05). The NMDS plot based on Bray–Curtis distance revealed substantial overlap between N+ (red dots) and N0 (blue dots) samples along NMDS1 and NMDS2, with no clear clustering or separation observed (PERMANOVA, *P* = 0.482) (**Figure 2C**). Similar results were obtained in validation cohort 1 (**Figures 2D–F**). Notably, in validation cohort 2, α-diversity differed significantly between groups, whereas β-diversity analysis still failed to show statistically significant separation (**Figures 2G–I**). These results suggest that overall microbial richness and community structure are largely similar between N0 and N+ groups, although subtle compositional differences may still be associated with LNM.

At the genus level, *Phascolarctobacterium* showed a higher detection rate and median relative abundance in N+ tumors than in N0 tumors. Specifically,*Phascolarctobacterium* was detected in 36 of 45 N+ tumors (80.0%) and 47 of 77 N0 tumors (61.04%), with median relative abundance values of 0.001128 and 0.0003639, respectively. The nominal difference was statistically significant (P = 0.003192),although it did not remain significant after FDR correction (FDR = 0. 1993). Complete genus-level detection rates, relative abundance summaries, nominal P values, and FDR-adjusted P values are provided in Online Resource 6. Sample-level *Phascolarctobacterium* relative abundance and detection status are provided in Online Resource 7.

LEfSe was then used as an exploratory genus-level screening method. Using a Kruskal–Wallis P < 0.05 and LDA score > 2.0, LEfSe identified 17 differentially enriched genera in the discovery cohort, including 14 genera enriched in the N+ group and 3 genera enriched in the N0 group (Online Resource 8). Among these taxa, Phascolarctobacterium was enriched in N+ tumors (LDA score > 3.0, P = 0.00314). As a compositionality-aware sensitivity analysis, ANCOM-BC2 showed a positive but non-significant association between Phascolarctobacterium abundance and N+ status (log-fold change = 0.320, P = 0.227, q = 0.435).

To further prioritize candidate taxa, LASSO/logistic regression modeling based on these LEfSe-screened genera retained two taxa with non-zero coefficients in the discovery cohort: Phascolarctobacterium (β = 0.095, odds ratio [OR] = 1.10) and Bacteroides (β = 0.009, OR = 1.01; Online Resource 9), suggesting a mild positive association of Phascolarctobacterium with N+ status.

In the two additional cohorts, Phascolarctobacterium showed directionally consistent enrichment in N+ tumors and was also retained in exploratory LASSO/logistic models. In additional cohort 1, Phascolarctobacterium had a positive coefficient (β = 0.157, OR = 1.17), and in additional cohort 2, it showed a similar positive direction (β = 0.080, OR = 1.08; Online Resources 8–9). These findings support cross-cohort directional consistency rather than formal validation of a pre-specified microbial model or threshold.

ROC analysis further showed that intratumoral *Phascolarctobacterium* abundance had moderate discriminative ability for lymph node metastasis (N+ vs N0), with the corresponding ROC curve and AUC shown in Online Resource 10.

Nevertheless, because ANCOM-BC2 did not identify Phascolarctobacterium as statistically significant after multiple-testing correction and β-diversity analyses did not show significant group separation, the primary microbiome signal should be regarded as modest and exploratory. Therefore, Phascolarctobacterium was carried forward for subsequent exploratory analyses of its clinical relevance in CRC.

### Prognostic value of *Phascolarctobacterium* in CRC

To reduce reliance on a dichotomized threshold, we first evaluated intratumoral Phascolarctobacterium abundance as a log-transformed continuous variable in a Cox proportional hazards regression model. In the discovery cohort, higher Phascolarctobacterium abundance was associated with worse overall survival (HR = 1.51, 95% CI: 1.00–2.27, P = 0.048).

**Figure 3:**
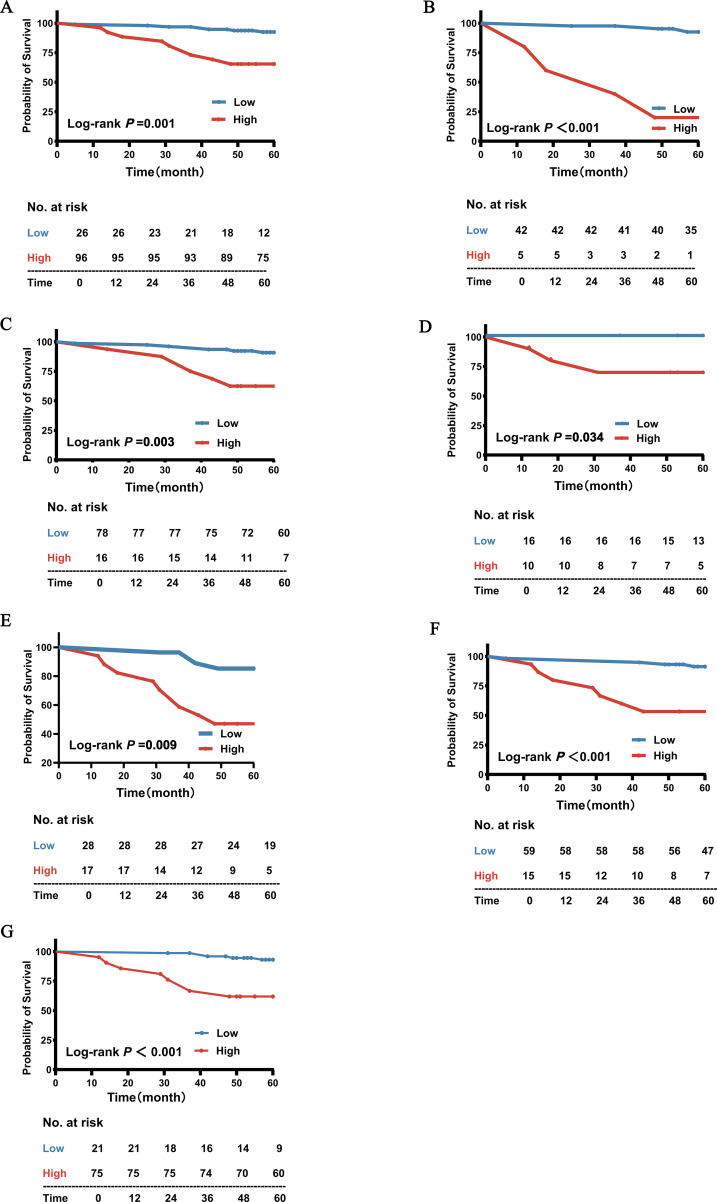
Prognostic association of Phascolarctobacterium abundance with overall survival in CRC. **(A)** Kaplan–Meier survival curves stratified by *Phascolarctobacterium* abundance (high vs low) in the discovery cohort using a ROC/Youden-derived exploratory threshold. Subgroup curves shown in panels B–G remained significant after Benjamini–Hochberg FDR correction for subgroup log-rank tests. **(B)** Stratified by age (>65 years). **(C, D)** Stratified by tumor size (≤5 cm vs >5 cm). **(E)** Stratified by lymph node status (N+ group). **(F)** Stratified by T stage (T3–T4 group). **(G)** Stratified by sex (male group). Red curves represent the high-abundance group; blue curves represent the low-abundance group.

Patients were then divided into high- and low-abundance groups using a ROC/Youden -derived cutoff defined within the discovery cohort (Online Resource 11). Kaplan– Meier survival analysis showed worse overall survival in the high-abundance group than in the low-abundance group (log-rank P < 0.05; **Figure 3A**). In Cox regression analysis based on this high/low abundance grouping, high Phascolarctobacterium abundance was associated with an increased risk of death in univariate analysis (HR = 5.105, 95% CI = 1.964–13.269, P < 0.001). N+ status (HR = 9.027, 95% CI = 2.589– 31.473, P < 0.001) and lower peripheral B-cell levels (HR = 0.822, 95% CI = 0.713– 0.948, P = 0.007) were also associated with overall survival. In multivariate Cox regression including these variables, high Phascolarctobacterium abundance remained associated with worse overall survival (HR = 3.892, 95% CI = 1.441–10.513, P = 0.007), together with N+ status and lower peripheral B-cell levels (**Table 2**).

**Table 2:** Exploratory Cox regression analyses of overall survival in the discovery cohort.

Variable	Level	Univariate HR (95% CI)	P	Multivariate HR (95% CI)	P
Phascolarctobacterium	Low	1.000		1.000	
	High	5.105 (1.964–13.269)	<0.001	3.892 (1.441–10.513)	0.007
N stage	N0	1.000		1.000	
	N+	9.027 (2.589–31.473)	<0.001	6.220 (1.754–22.055)	0.005
Age (years)	≤65	1.000			
	>65	1.453 (0.560–3.772)	0.442		
Tumor size (cm)	≤5	1.000			
	>5	0.848 (0.244–2.954)	0.796		
Sex	Female	1.000			
	Male	2.108 (0.686–6.471)	0.193		
T stage	T1–T2	1.000			
	T3–T4	1.220 (0.350–4.250)	0.754		
T cell (%)		0.976 (0.936–1.018)	0.258		
B cell (%)		0.822 (0.713–0.948)	0.007	0.826 (0.707–0.965)	0.016
CA19-9 (U/mL)		0.996 (0.970–1.024)	0.793		
CEA (ng/mL)		0.905 (0.760–1.078)	0.265		

CRC, colorectal cancer; N0, lymph node metastasis-negative; N+, lymph node metastasis-positive; tumor size: maximum tumor diameter; HR, hazard ratio; CI, confidence interval.

Variables with P < 0.05 in univariate analysis were entered into the multivariate Cox regression model. Phascolarctobacterium abundance was analyzed using an exploratory ROC/Youden-derived high/low grouping. Survival analyses were performed in the discovery cohort. Log-transformed Phascolarctobacterium abundance was additionally analyzed as a continuous variable to reduce reliance on dichotomization.

Subgroup Kaplan–Meier analyses were performed to assess whether the prognostic trend was observed across clinical strata. After Benjamini–Hochberg FDR correction for subgroup log-rank tests, significant survival differences remained in male patients, patients aged >65 years, patients with tumor size ≥5 cm or ≤5 cm, N+ patients, and patients with T3–T4 tumors; these FDR-significant subgroup curves are shown in **Figures 3B–G**. Survival differences in the remaining subgroups did not reach statistical significance after FDR correction. Kaplan–Meier curves for the remaining subgroups are provided in Online Resource 12, and complete subgroup statistics, including sample sizes, event numbers, raw log-rank P values, and FDR-adjusted P values, are provided in Online Resource 13.

We further assessed the stability of the ROC signal and the Youden-derived cutoff using 1,000 bootstrap resampling iterations. The bootstrap mean AUC was 0.667, with a 95% range of 0.469–0.819, indicating moderate but variable discriminative ability. The estimated cutoff varied across resampled datasets.

To evaluate whether the prognostic association was influenced by nodal status, we fitted a Cox model including log-transformed Phascolarctobacterium abundance, N stage, and their interaction term. The interaction term was not statistically significant (HR = 1.33, 95% CI = 0.54–3.25, P = 0.535), suggesting no evidence that this association differed significantly between N0 and N+ patients.

Overall, these results suggest that higher intratumoral Phascolarctobacterium abundance is associated with adverse prognosis in the discovery cohort. Because the survival cutoff was derived from the same cohort, the number of events was limited, and independent survival validation was not available, these findings should be interpreted as exploratory and require further validation in larger independent cohorts.

### Association of *Phascolarctobacterium* with tumor immune cell infiltration

Given the limited taxonomic resolution of 16S rRNA sequencing, we performed BLAST analysis for each ASV assigned to the genus Phascolarctobacterium to further refine taxonomic annotation. Most ASVs showed high sequence similarity to Phascolarctobacterium succinatutens, supporting this species as the predominant representative within this genus in our dataset. On this basis, Phascolarctobacterium succinatutens was selected for subsequent FISH analysis.

**Figure 4:**
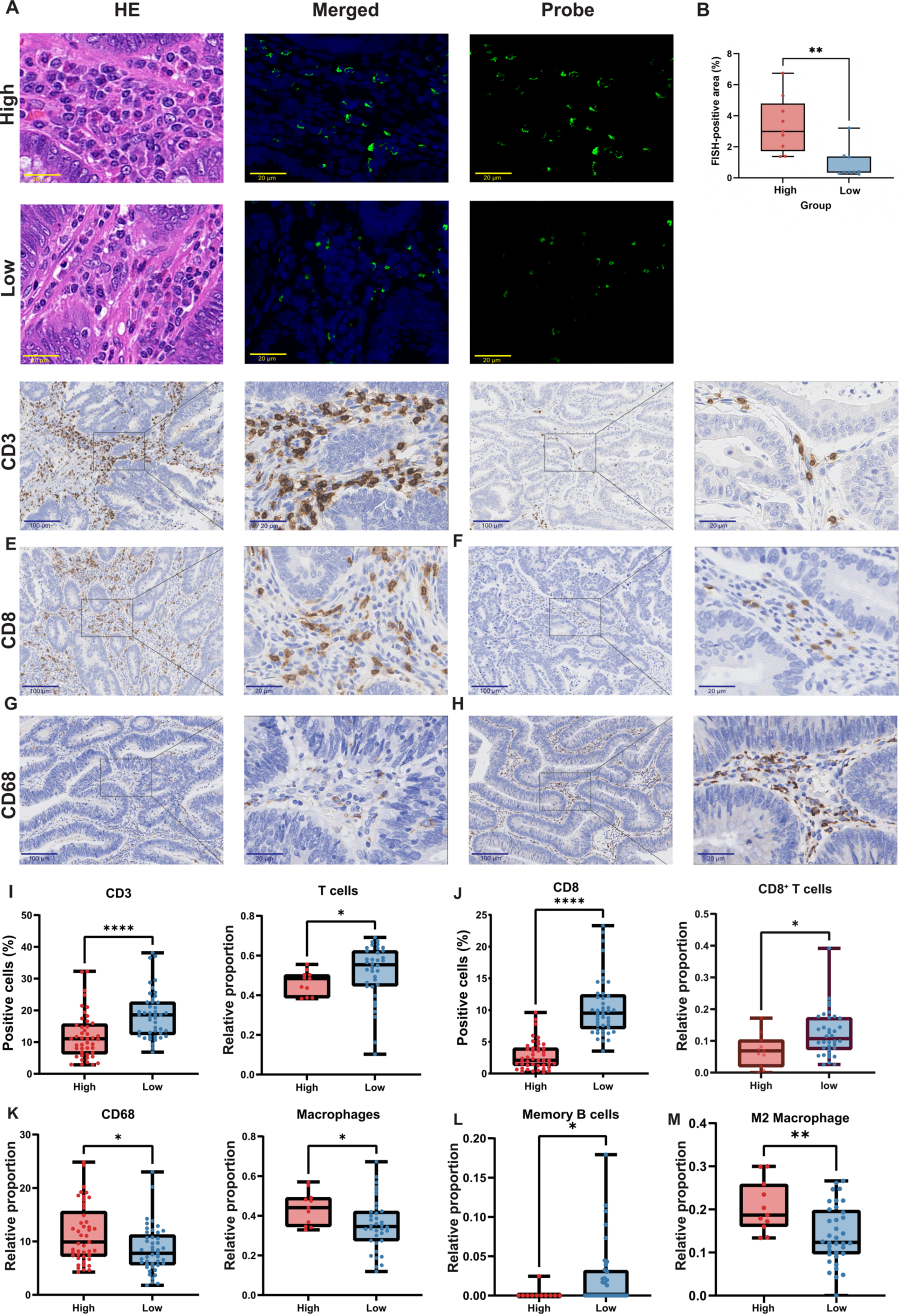
Association between *Phascolarctobacterium* abundance and immune- contexture features. **(A)** Representative HE and high-magnification FISH images from the high- and low-Phascolarctobacterium-abundance groups. Merged images show DAPI nuclear counterstaining and FITC-labeled Phascolarctobacterium succinatutens-like probe signals. Probe-specific single-channel images are shown in the main figure, and corresponding DAPI single-channel images are provided in Online Resource 18 to allow evaluation of unmerged fluorescence channels. **(B)** Quantification of FISH-positive area fraction. FISH-positive area fraction was calculated as the green-channel FISH-positive area divided by the annotated tumor ROI area. Quantification was performed using three representative tumor fields from each of three individual tumor samples per group. **(C)** Representative CD3 IHC staining in the low-Phascolarctobacterium-abundance group. **(D)** Representative CD3 IHC staining in the high-Phascolarctobacterium-abundance group. **(E)** Representative CD8 IHC staining in the low-Phascolarctobacterium-abundance group. **(F)** Representative CD8 IHC staining in the high-Phascolarctobacterium-abundance group. **(G)** Representative CD68 IHC staining in the low-Phascolarctobacterium-abundance group. **(H)** Representative CD68 IHC staining in the high-Phascolarctobacterium-abundance group. **(I–K)** Quantification of CD3, CD8, and CD68 IHC staining based on positive cell percentages within annotated tumor ROIs, together with corresponding CIBERSORT-derived immune-cell estimates. IHC staining was performed in 30 CRC tumor samples, including 15 Phascolarctobacterium-high and 15 Phascolarctobacterium-low tumors, with three representative high-power tumor fields analyzed per sample for each marker. **(L, M)** CIBERSORT-derived estimates for memory B cells and M2 macrophages. Definitions: Total T cells = CD4 subsets + γδT + CD8; Total macrophages = M0 + M1 + M2. Statistical tests: Shapiro–Wilk for normality; Welch’s t-test if normal; Wilcoxon rank-sum if non-normal. Significance: *P < 0.05, **P < 0.01, ***P < 0.001, ****P < 0.0001.

Species-specific FISH was performed to provide spatial support for the sequencing-derived Phascolarctobacterium signal in tumor tissue sections. Punctate Phascolarctobacterium succinatutens-like FISH signals were observed within pathologist-reviewed colorectal adenocarcinoma regions in representative samples from both high- and low-abundance groups (**Figure 4A**). Image-based quantification using QuPath showed that the green-channel FISH-positive area fraction was higher in the high-abundance group than in the low-abundance group (**Figure 4B**). Quantification was performed using three representative tumor fields from each of three individual tumor samples per group. These findings provide supportive histological evidence for tumor tissue-associated Phascolarctobacterium-like signals, although they should not be interpreted as definitive evidence of viable bacterial colonization, intracellular localization, or bacterial–immune-cell co-localization.

To assess the tumor immune contexture, IHC staining for CD3, CD8, and CD68 was performed in 30 tumor samples, including 15 Phascolarctobacterium-high and 15 Phascolarctobacterium-low tumors. For each marker, three representative high-power tumor fields were analyzed per sample within pathologist-reviewed tumor regions. Representative IHC images are shown in **Figures 4C–H**, and the corresponding IHC quantification is shown in **Figures 4I–K**. IHC analysis showed greater infiltration of CD3+ T cells and CD8+ T cells in the low-abundance group, whereas CD68+ macrophages were more abundant in the high-abundance group.

CIBERSORT deconvolution of bulk RNA-seq data was performed to provide a transcriptome-based estimate of immune-cell composition and to complement the histological information provided by IHC. Consistently, CIBERSORT showed higher proportions of total T cells and CD8+ T cells in the low-abundance group, together with higher estimated proportions of total macrophages, particularly M2 macrophages, in the high-abundance group; memory B cells were also elevated in the low-abundance group (**Figures 4I–M**). Together, these results suggest that higher Phascolarctobacterium abundance is associated with distinct immune-contexture features, including reduced T-cell-related signals and increased macrophage-related signals. These findings should be interpreted as exploratory associations rather than evidence of direct immune regulation.

### Phascolarctobacterium-associated gene modules and pathways

**Figure 5:**
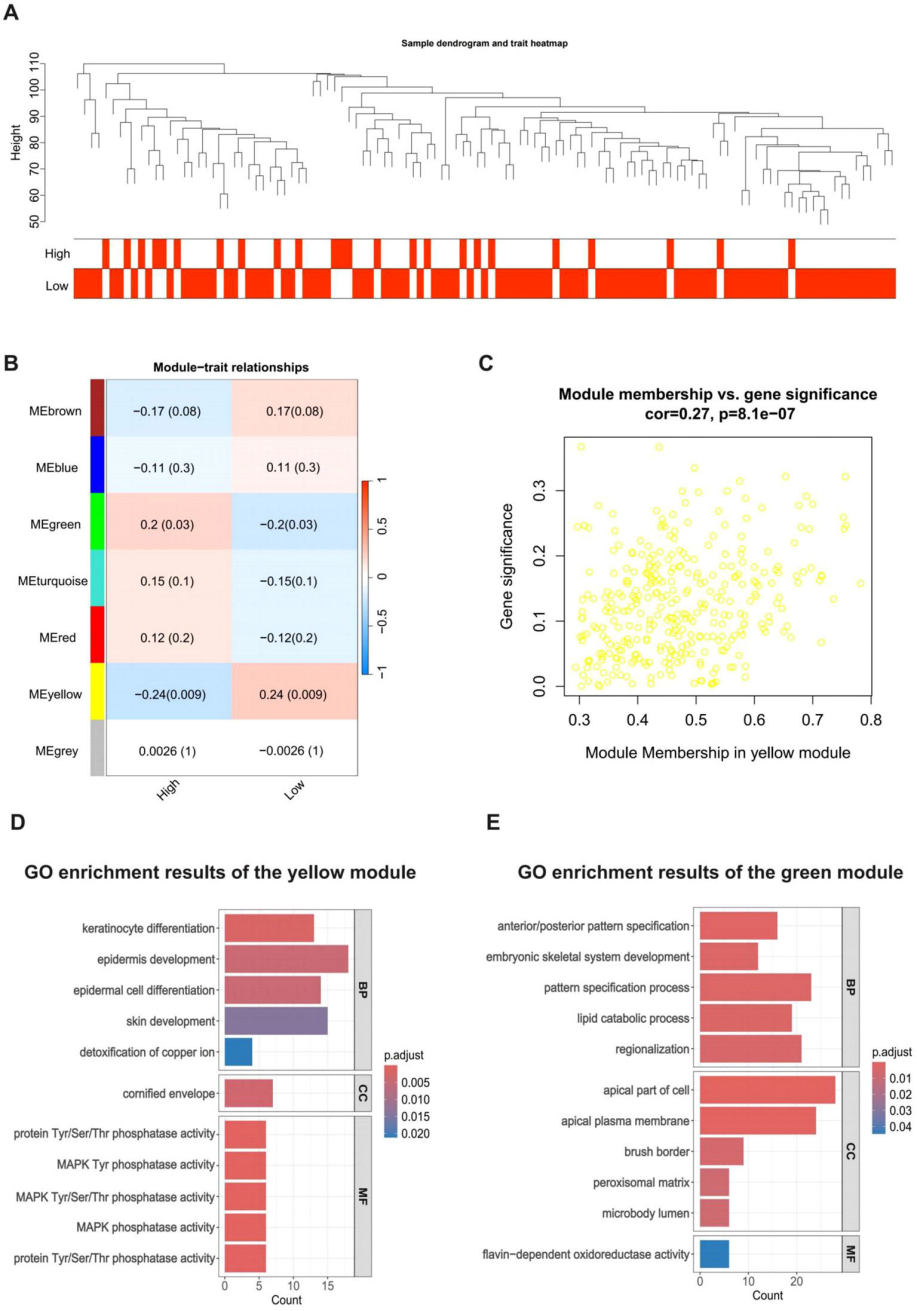
Exploratory WGCNA module identification and functional enrichment analysis. **(A)** Sample dendrogram and trait heatmap after hierarchical clustering. **(B)** Module–trait correlation heatmap between module eigengenes and *Phascolarctobacterium* abundance. **(C)** Scatter plot of module membership vs gene significance for the yellow module. **(D)** GO enrichment of the yellow module. **(E)** GO enrichment of the green module.

In the discovery cohort, WGCNA was applied to construct gene co-expression networks. First, sample filtering was performed based on the sample clustering tree at a height threshold of ll0 and the co-expression network was subsequently constructed using the filtered samples (**Figure 5A**). A soft-thresholding power of β = 5 was then selected to approximate scale-free topology (Online Resource 14).

Correlation analysis of module eigengenes with microbial abundance identified two significantly associated modules: the yellow module, which was positively correlated with the low-abundance group (r = 0.24, P = 0.009), and the green module, which was positively correlated with the high-abundance group (r = 0.20, P = 0.03) (**Figure 5B**).

Further analysis revealed that hub genes in the yellow module showed a stronger correlation with Phascolarctobacterium abundance (r = 0.27, P < 0.001), whereas the correlation between hub genes in the green module and Phascolarctobacterium abundance was relatively weak (r = 0.13, P = 0.035) (**Figure 5C**; Online Resource 14).

Gene Ontology (GO) and Kyoto Encyclopedia of Genes and Genomes (KEGG) enrichment analyses revealed that the yellow module was mainly enriched in metabolic and antioxidant-related pathways, including lipid catabolic process, organic acid transport, oxidoreductase activity, Peroxisome proliferator–activated receptor (PPAR) signaling, peroxisome, and Wingless/Integrated (Wnt) signaling. In contrast, the green module was predominantly enriched in pathways related to epithelial barrier function and stress responses, such as keratinocyte differentiation, epidermis development, cornified envelope formation, and mitogen-activated protein kinase (MAPK) signaling (**Figures 5D, E**). For readability, only representative enriched terms are shown in **Figures 5D, E**, and the complete GO and KEGG enrichment results are provided in Online Resource 15.

## Discussion

To our knowledge, this study provides exploratory evidence that tumor tissue-associated Phascolarctobacterium abundance—predominantly represented by Phascolarctobacterium succinatutens based on BLAST annotation and supported by species-specific FISH-like signals—shows a modest association with lymph node metastasis (LNM) and poorer overall survival in the discovery cohort of colorectal cancer (CRC). By integrating immunohistochemistry (IHC) and transcriptomic deconvolution analyses, we observed patient-level associations between higher Phascolarctobacterium abundance and immune-contexture features, including higher macrophage/M2 macrophage-related signals and lower CD3+ and CD8+ T-cell-related signals. Collectively, these findings should be interpreted as exploratory associations rather than evidence of direct microbial immune modulation or metastatic causation.

Although β-diversity analyses did not reveal significant global separation between LNM-positive and LNM-negative tumors, this finding indicates that the overall intratumoral microbial community structure is largely conserved across groups. Importantly, the absence of community-level divergence does not preclude biologically meaningful associations at the level of individual taxa, as β-diversity metrics assess global community dissimilarity, whereas differential abundance analyses focus on taxon-specific signals. In this context, differential abundance analyses suggested a directionally consistent increase in Phascolarctobacterium in LNM-positive tumors across the discovery cohort, the internal validation cohort, and the external validation cohort, supporting a modest taxon-level association rather than a broad microbiome shift. Similar patterns have been described in tumor-associated microbiome studies, in which discrete microbial taxa are linked to tumor progression or immune modulation without overt restructuring of the entire microbial community (McMurdie and Holmes, 2014; Wirbel et al., 2019).

Recent clinical and multi-omics investigations provide important context for our findings. Circulating immune profiling in colorectal cancer has revealed systemic immune alterations associated with disease progression, underscoring a strong link between immune composition and patient prognosis (Guan et al., 2024). In parallel, large-scale transcriptomic analyses of lymph nodes have demonstrated pronounced heterogeneity, characterized by distinct immune and stromal programs, indicating that nodal biology is tightly integrated with tumor immunity and clinical outcomes (Guan et al., 2025). Moreover, studies in patients with colorectal cancer liver metastasis have shown that lymph node metastasis confers adverse prognosis and is accompanied by immune-microenvironmental remodeling, highlighting the clinical relevance of LNM–immunity interactions (Zhang et al., 2025). Taken together, these reports support a framework in which tumor-associated immune states and nodal ecosystems are closely interconnected, providing a biological context in which tumor tissue-associated microbial features may correlate with nodal status and local immune contexture.

From a microbiological perspective, Phascolarctobacterium is a strictly anaerobic, Gram-negative genus within the family Acidaminococcaceae and a common constituent of the human gut microbiota. It is primarily characterized as a succinate- utilizing bacterium involved in microbial metabolic cross-feeding, converting succinate into short-chain fatty acids such as propionate. Previous studies have linked Phascolarctobacterium abundance to host metabolic and inflammatory states, suggesting preferential adaptation to specific metabolic niches rather than a classical pathogenic role. In colorectal cancer, species-resolved and tissue-based microbiome studies have reported enrichment of Phascolarctobacterium in tumor-associated microbial communities. In addition, a recent study of fecal microbial dysbiosis in CRC also identified Phascolarctobacterium as associated with colorectal cancer risk, providing further external support for a CRC-related association of this genus (Kim et al., 2025). However, its functional role within the tumor microenvironment remains incompletely defined, and the current evidence is still largely associative.

In addition to Phascolarctobacterium, we observed differential abundance of several other tumor-associated microbial taxa, including Actinomyces, Desulfovibrio, and Bacteroides, findings that are consistent with previously reported body mass index–associated microbial patterns in colorectal cancer patients (Huang et al., 2024). While prior mechanistic studies have demonstrated that certain bacteria, such as Campylobacter jejuni, can directly promote colorectal cancer metastasis through defined signaling pathways, including the JAK2–STAT3–MMP9 axis (He et al., 2024), the present study does not establish a causal role for Phascolarctobacterium. Rather, our results suggest that multiple tumor tissue-associated microbial taxa may correlate with metastatic phenotypes, potentially reflecting adaptation to tumor-associated immune and metabolic microenvironments.

WGCNA further suggested exploratory associations between Phascolarctobacterium abundance and gene modules linked to epithelial stress responses, such as MAPK signaling and barrier remodeling, as well as metabolic regulation, including lipid metabolism and PPAR signaling. These patterns indicate correlation-based host transcriptomic differences associated with Phascolarctobacterium abundance. However, because WGCNA was exploratory and not fully adjusted for tumor purity, batch effects, or clinical covariates, these results should not be interpreted as evidence that Phascolarctobacterium directly regulates host pathways. Such integrative network-based approaches have been successfully used to link microbiome and host transcriptomic or metabolomic modules in cancer and other diseases (Guan et al., 2023).

Large-scale microbiome studies have shown that microbial signatures may provide disease-related information in colorectal cancer. In the present study, Phascolarctobacterium abundance showed an exploratory association with poorer overall survival in the discovery cohort; however, whether it provides incremental prognostic information beyond conventional Tumor–Node–Metastasis (TNM) staging remains unknown and requires validation in larger independent cohorts. Although cutoff-based Kaplan–Meier analysis showed a significant survival difference, additional sensitivity analyses suggested that the prognostic association was not solely dependent on dichotomization by a single threshold. In particular, the association remained significant when Phascolarctobacterium abundance was modeled as a log-transformed continuous variable. At the same time, bootstrap analyses indicated that while the ROC signal was moderate overall, the Youden-derived cutoff showed variability across resampled datasets. Therefore, the cutoff used in the present study should be considered data-driven and exploratory rather than definitive, and further validation in independent cohorts is required before any fixed threshold can be regarded as clinically robust. At present, therapeutic implications of Phascolarctobacterium remain speculative and are not supported by direct functional evidence in CRC.

Moreover, integrating microbial abundance with immune-related variables may be worth exploring in future prognostic models, although its clinical utility remains to be established.

Several limitations should be acknowledged. First, the single-center, retrospective design may introduce selection bias and confounding factors; although one internal and one external validation cohort were included, multi-center prospective studies are warranted.

Second, 16S rRNA sequencing limits resolution to the genus level, restricting functional insights at the strain or subspecies level; metagenomic or metatranscriptomic sequencing could address this limitation. Third, our results are primarily correlative and pathway-based, lacking direct functional validation; future studies should employ organoid co-culture systems or germ-free animal models to establish causality. In addition, the survival cutoff used for dichotomizing Phascolarctobacterium abundance was derived within the discovery cohort and showed some variability in bootstrap analyses. Therefore, this threshold should be interpreted cautiously and requires validation in independent cohorts.

In addition, because 16S rRNA sequencing was performed on bulk tumor tissues, the detected bacterial profiles should be interpreted as tumor tissue-associated microbial signals rather than definitive evidence of viable intratumoral colonization. Although negative controls and decontamination procedures were applied, we cannot fully exclude contamination introduced during tissue handling, sectioning, or pathological processing. We did not perform total bacterial load quantification or Phascolarctobacterium-specific qPCR/digital PCR validation; therefore, relative-abundance findings require further confirmation using quantitative and spatially resolved approaches.

Furthermore, IHC analysis was performed on an available tissue-microarray subset and was not fully matched one-to-one with the 16S rRNA sequencing cohort.

Paired bacterial FISH and immune-marker co-staining was not performed; therefore, the immune-related findings should be interpreted as patient-level associations rather than evidence of bacterial–immune-cell spatial co-localization or direct immune regulation. WGCNA was exploratory and was not fully adjusted for tumor purity, batch effects, or clinical covariates, which limits mechanistic interpretation.

Future studies should prioritize quantitative validation of Phascolarctobacterium using total 16S rRNA, genus/species-specific qPCR or digital PCR, spatial co-localization analyses combining bacterial FISH with immune markers, and functional experiments in organoid or animal models. Metagenomic and metabolomic approaches will also be needed to determine whether Phascolarctobacterium-related signals reflect viable bacteria, bacterial products, or broader tumor-associated microbial and metabolic states.

## Data Availability

The raw data supporting the conclusions of this article will be made available by the authors, without undue reservation.
