# Supplementary material for: Tumor tissue-associated Phascolarctobacterium is associated with lymph node metastasis, prognosis, and immune-contexture features in colorectal cancer
Source: Front Cell Infect Microbiol. 2026 Jul 8;16:1784151. doi: 10.3389/fcimb.2026.1784151 (PMC13388136; doi:10.3389/fcimb.2026.1784151)
Supplement: Supplementary file 1 [file DataSheet1.pdf]

1 Supplementary Figures

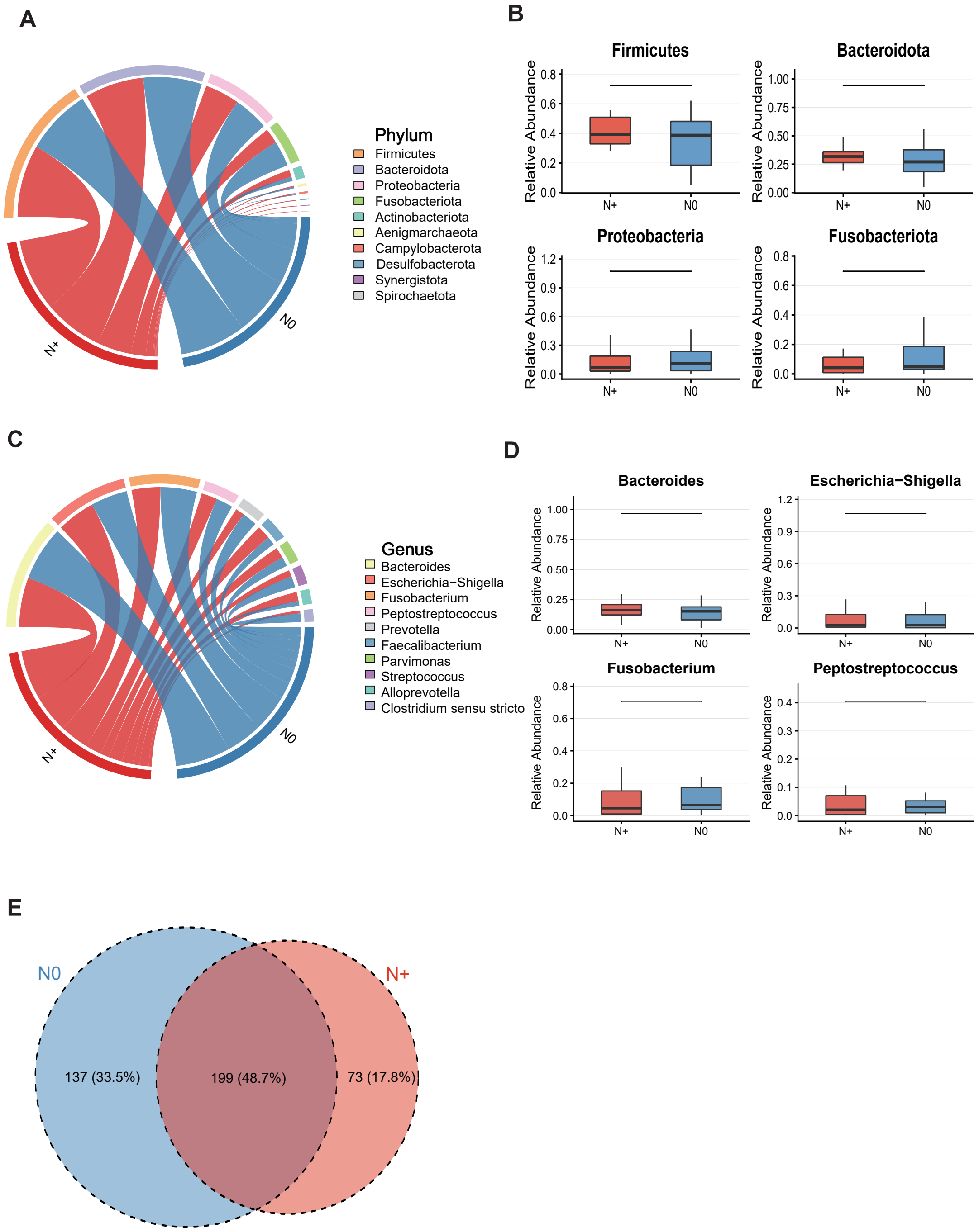

Online Resource 4 : Analysis of differences in gut microbiota composition and abundance between CRC patients with N+ and N0 in validation cohort 1.

Abbreviations: CRC, colorectal cancer; N0, lymph node metastasis-negative; N+, lymph node metastasis-positive; F/B ratio, Firmicutes/Bacteroidota ratio.

(A) Phylum-level chord diagram. (B) Boxplots of major phyla; Bacteroidota enriched in N+. (C) Genus-level chord diagram. (D) Boxplots of major genera; Bacteroides increased in N+. (E) Venn diagram showing shared and unique genera.

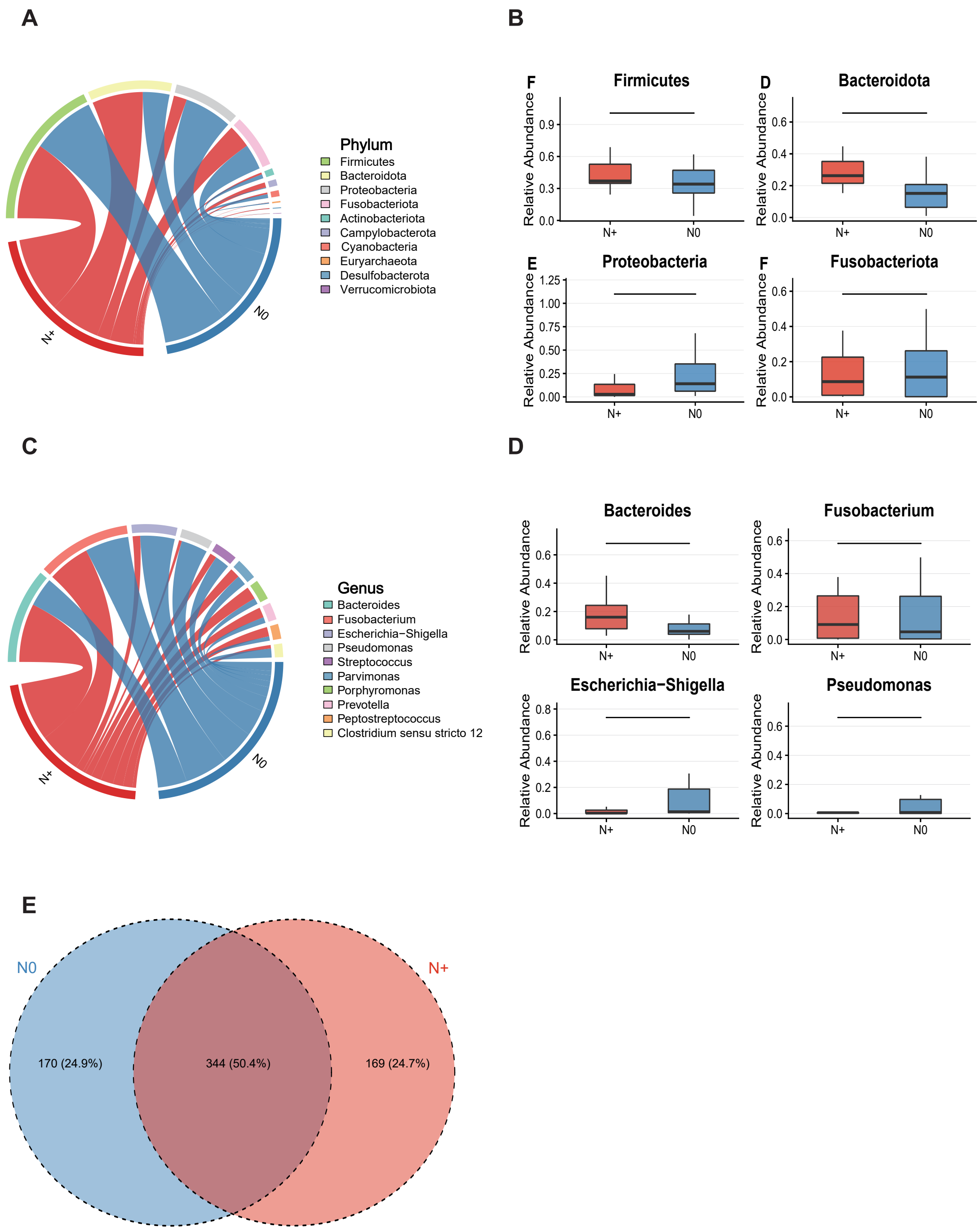

Online Resource 5 : Analysis of differences in gut microbiota composition and abundance between CRC patients with N+ and N0 in validation cohort 2.

Abbreviations: CRC, colorectal cancer; N0, lymph node metastasis-negative; N+, lymph node metastasis -positive; F/B ratio, Firmicutes/Bacteroidota ratio.

(A) Phylum-level chord diagram. (B) Boxplots of major phyla; Bacteroidota enriched in N+. (C)Genus-level chord diagram. (D) Boxplots of major genera; Bacteroides increased in N+. (E) Venn diagram showing shared and unique genera.

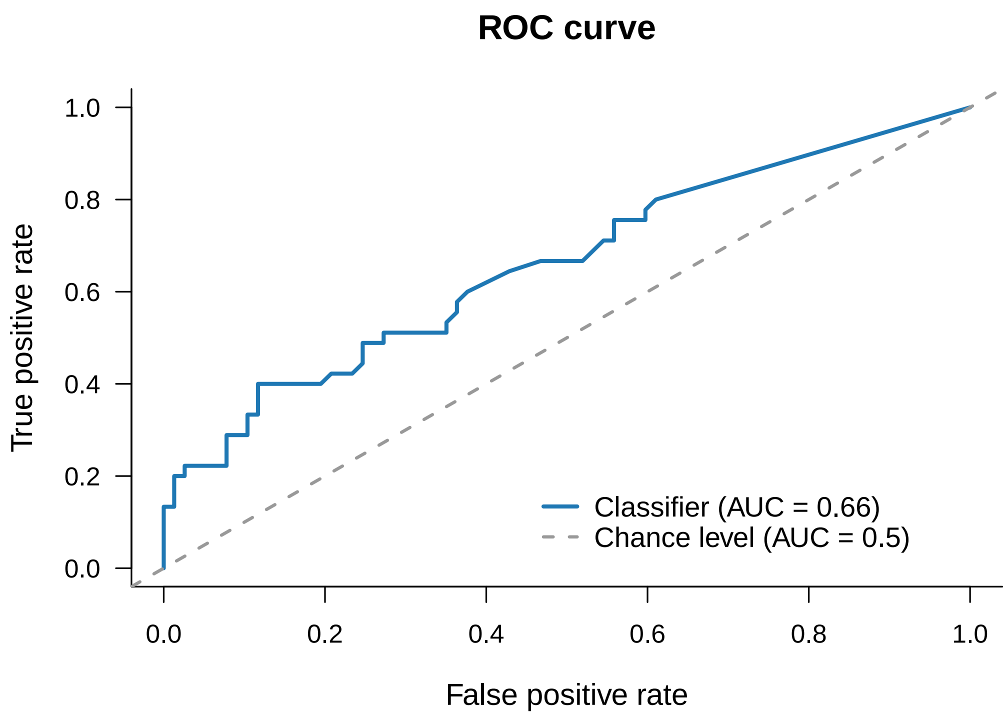

Online Resource 1 0 : Receiver operating characteristic (ROC) analysis of intratumoral *Phascolarct oba cterium* abundance for discriminating lymph node metastasis in CRC patients. The ROC curve ev alu ates the ability of intratumoral *Phascolarctobacterium* abundance to distinguish lymph node metast asis -positive (N+) from lymph node metastasis-negative (N0) cases, with the corresponding area und er t he curve (AUC) indicated.

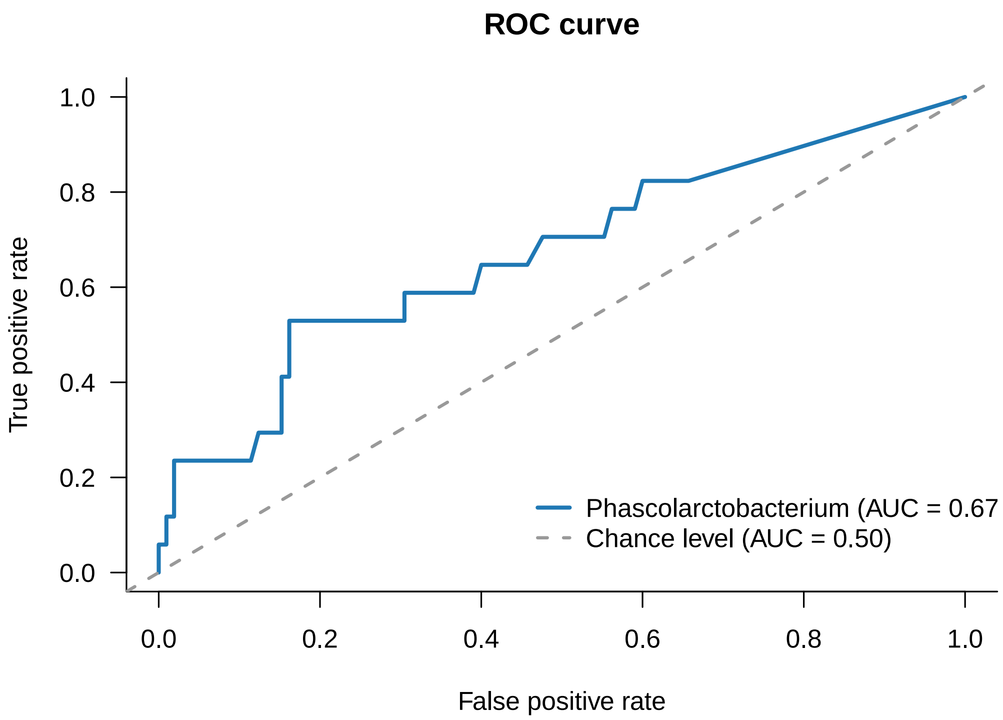

Online Resource 1 1 : Receiver operating characteristic (ROC) analysis of intratumoral *Phascolarctob acterium* abundance for overall survival stratification in colorectal cancer. The ROC curve was genera ted using survival status at the last follow-up (deceased vs alive) as the outcome to evaluate the ability of intratumoral *Phascolarctobacterium* abundance to discriminate survival status. The area unde r the curve (AUC) is shown, and the optimal cutoff value was determined using the Youden index for subsequent survival analyses.

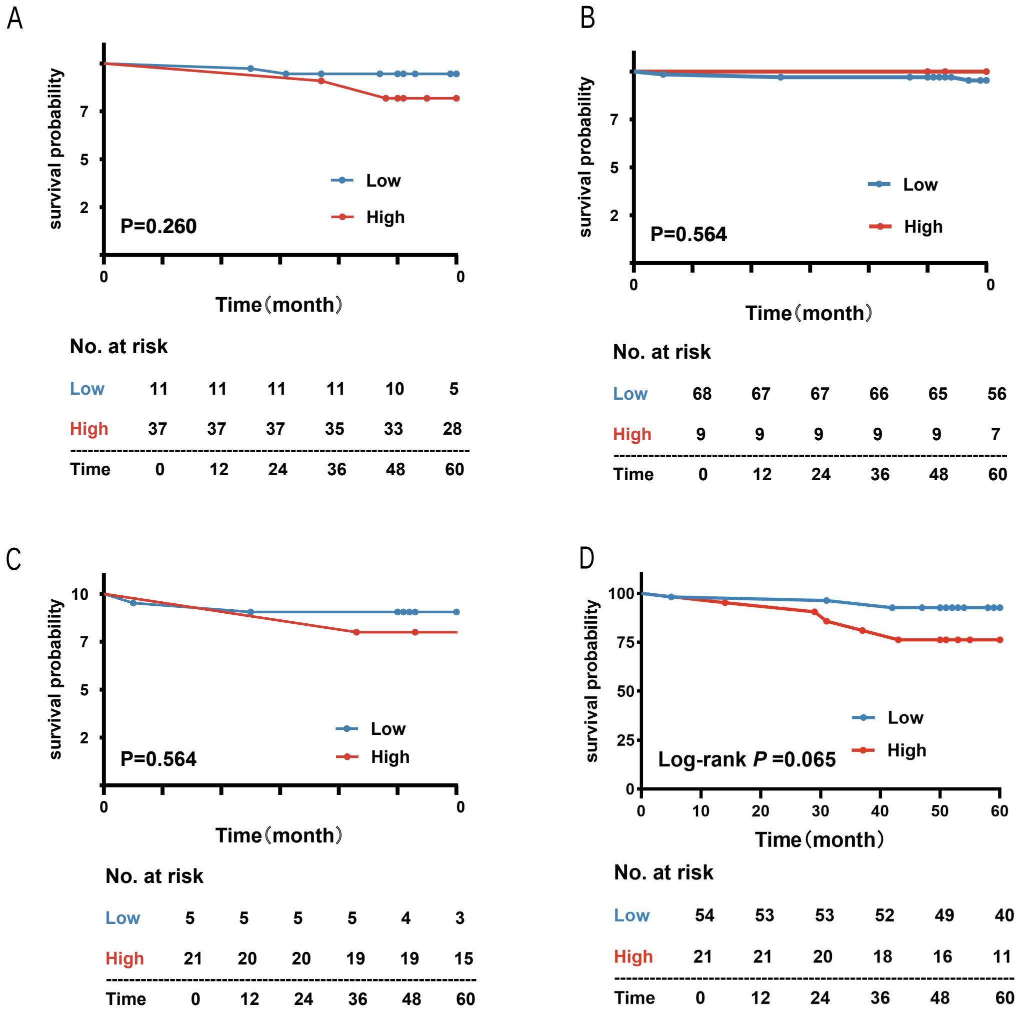

Online Resource 1 3 : Prognostic value of *Phascolarctobacterium* and clinical characteristics in CRC patients .**Abbreviations:** CRC, colorectal cancer; N0, lymph node metastasis-negative; N+, lymph node metastasis-positive; tumor size: maximum tumor diameter.

(A) Stratified by sex (female group). (B) Stratified by lymph node status (N0 group). (C) Stratified by T stage (T1–T2 group). (D) Stratified by age (≤65 group)

Survival differences were compared using the log-rank test. Red lines indicate the high-expression group, and blue lines represent the low-expression group.

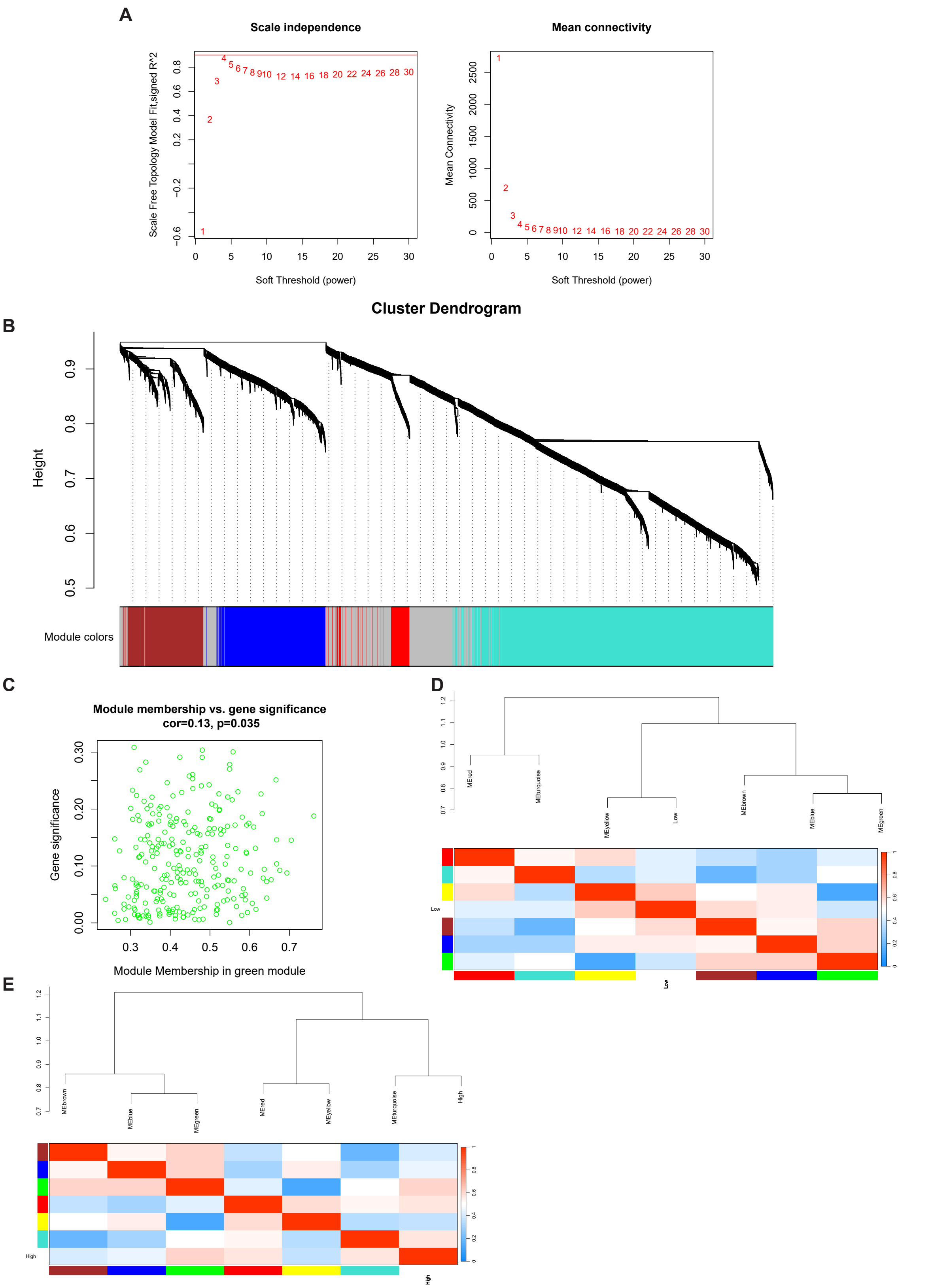

Online Resource 1 4 : Weighted gene co-expression network analysis (WGCNA) of tumor transcriptom e data.

Abbreviations: WGCNA, weighted gene co-expression network analysis; GO, Gene Ontology; ME, module eigengene; GS, gene significance. (A) Scale-free topology fit index and mean connectivity across soft-thresholding powers. (B) Hierarchical clustering dendrogram showing gene modules (colored by module). (C) Correlation between module membership and gene significance in the turquoise module. (D) Correlation heatmap between low–*Phascolarctobacterium* abundance and module eigengenes. (E) Correlation heatmap between high–*Phascolarctobacterium* abundance and module eigengenes. The color scale represents correlation strength and direction (red, positive; blue, negative).

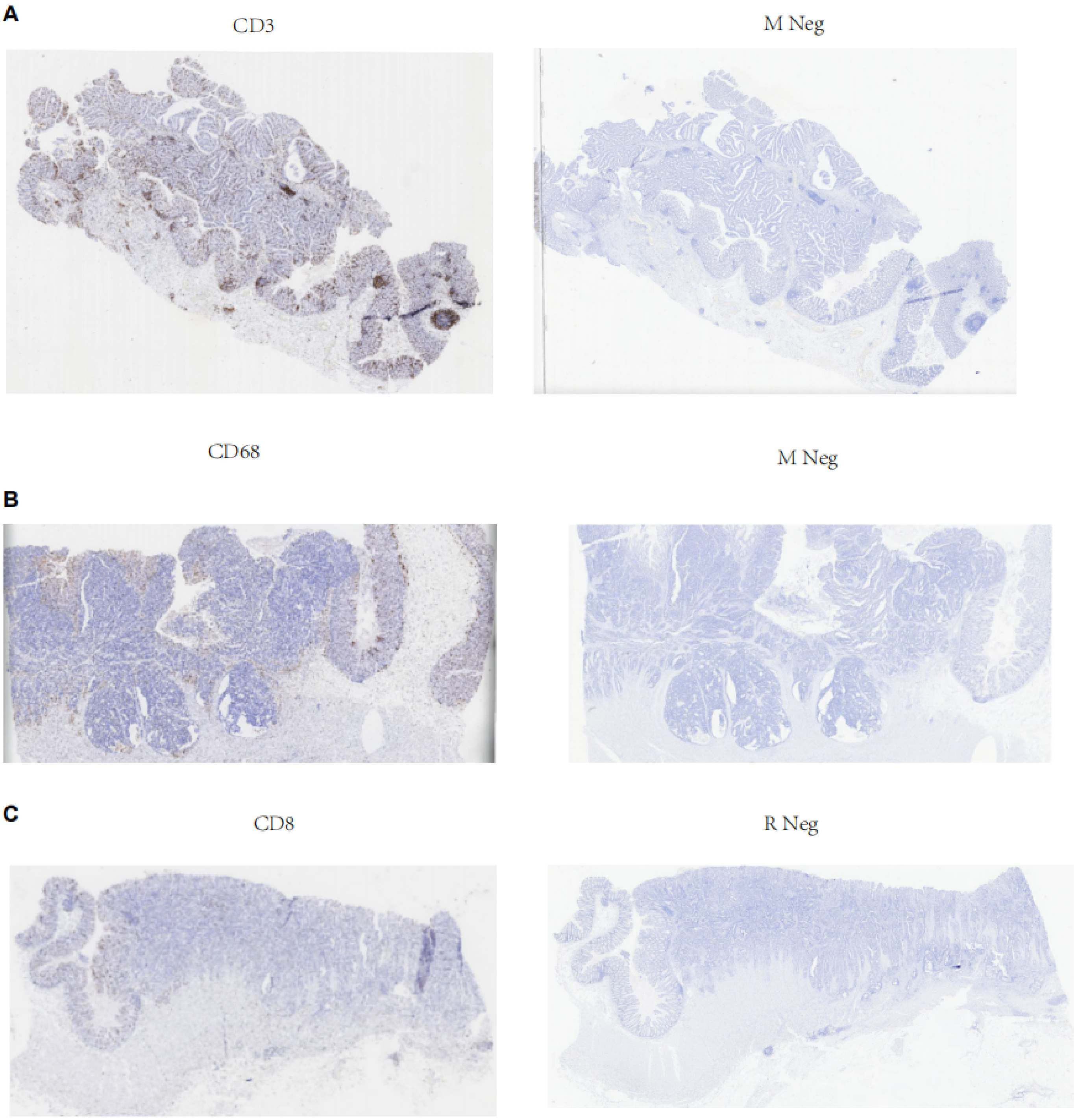

Online Resource 1 6 :Representative immunohistochemical staining of CD3, CD68 and CD8with matched IgG negative controls.

A, CD3 staining and mouse IgG negative control. B, CD68 staining and mouse IgG negative control.

C, CD8 staining and rabbit IgG negative control. Brown signals indicate positive DAB staining, and nuclei were counterstained with hematoxylin. The IgG controls showed minimal background staining and no evident specific immunoreactivity.

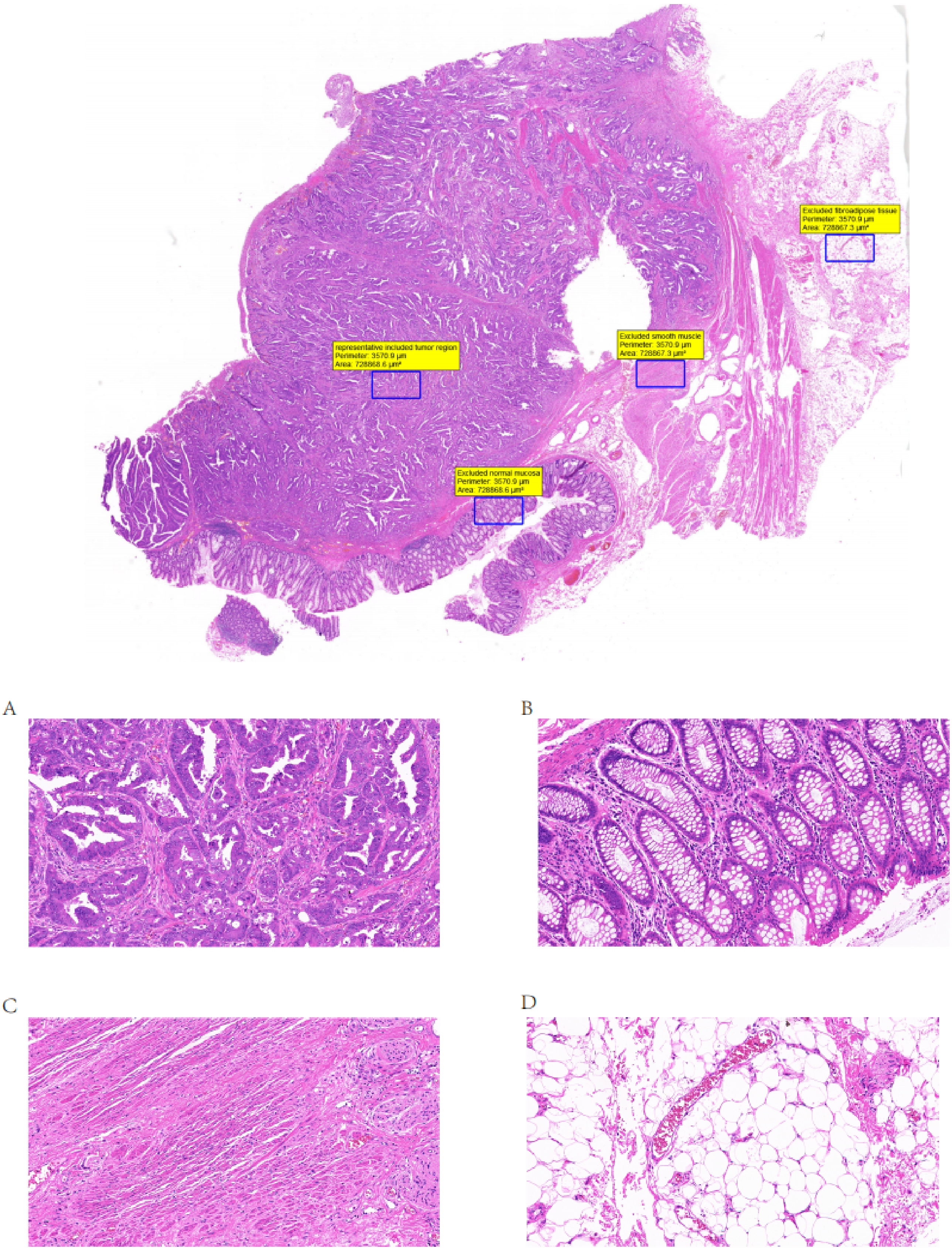

Online Resource 1 7 :Representative HE-stained image showing tumor-region selection.

A, included tumor region; B, excluded normal mucosa/lamina propria;C, excluded smooth muscle; D, excluded fibroadipose tissue. Quantification of IHC-positive cells and FISH-positive signals was restricte d to pathologist-reviewed tumor regions, while necrotic and non-tumor areas were excluded.

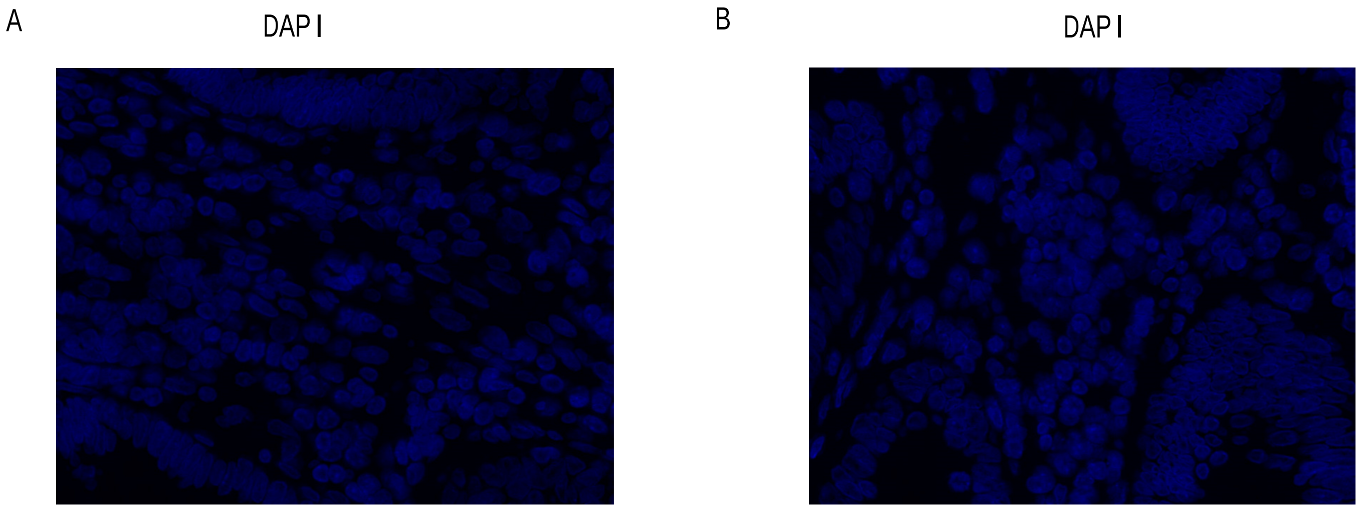

Online Resource 18: DAPI single-channel images corresponding to Fig. 4A.
(A) Phascolarctobacterium-high group.
(B) Phascolarctobacterium-low group.
